# Evaluation of the E-PASS, mE-PASS, and POSSUM scoring systems for predicting postoperative complications following radical nephrectomy

**DOI:** 10.1186/s12893-026-03960-7

**Published:** 2026-06-16

**Authors:** Umit Uysal, Ergun Alma, Adem Altunkol, Hakan Anıl, Kadir Karkin, Mert Hamza Özbilen, Cafer Tayyer Akçor, Yusuf Enes Kök, Buğra Aksay

**Affiliations:** Department of Urology, Health Sciences University Adana City Training and Research Hospital, Adana, 01000 Turkey

**Keywords:** E-PASS, POSSUM, Postoperative complications, Radical nephrectomy Surgical scoring system

## Abstract

**Background:**

Accurate prediction of postoperative complications following radical nephrectomy is of great clinical importance for perioperative risk stratification and patient management. Although several scoring systems are used in urological surgery, there is no widely accepted, procedure-specific risk assessment tool. This study aimed to evaluate the performance of the Physiological and Operative Severity Score for the Enumeration of Mortality and Morbidity (POSSUM), Estimation of Physiologic Ability and Surgical Stress (E-PASS), and its modified version (mE-PASS) in predicting postoperative complications after radical nephrectomy. As a secondary objective, the association between modified Glasgow Prognostic Score (mGPS), Prognostic Nutritional Index (PNI), and neutrophil-to-lymphocyte ratio (NLR), which reflect systemic inflammation and nutritional status, and postoperative complications was also investigated.

**Materials and methods:**

A total of 148 patients who underwent radical nephrectomy for renal tumors at our institution between January 2022 and March 2025 were retrospectively analyzed. Patients were divided into two groups based on the presence of postoperative complications. Postoperative complications were assessed according to the Clavien–Dindo classification, and complications of grade ≥ 2 were included in the analysis. Demographic characteristics, comorbidities, preoperative laboratory parameters, surgical variables, and postoperative outcomes were recorded. The predictive performance of the scoring systems was evaluated using the area under the receiver operating characteristic (ROC) curve (AUC).

**Results:**

Postoperative complications were observed in 51 patients (34.5%). The POSSUM score demonstrated the highest predictive performance with an AUC of 0.810, showing high sensitivity (82.4%) and specificity (71.1%). The E-PASS score was also statistically significant but exhibited a more limited predictive performance (AUC: 0.682). In contrast, the mE-PASS score showed markedly lower predictive ability (AUC: 0.599). Patients who developed complications had significantly lower PNI values and higher NLR values (*p* < 0.05). Additionally, intraoperative blood loss, tumor size, and length of hospital stay were significantly greater in the complication group (*p* < 0.05).

**Conclusion:**

POSSUM and E-PASS may serve as useful tools for predicting postoperative complications following radical nephrectomy. However, given their moderate predictive performance and the limitations of the present study, these findings should be validated in larger, prospective, multicenter studies.

**Supplementary Information:**

The online version contains supplementary material available at 10.1186/s12893-026-03960-7.

## Introduction

Radical nephrectomy remains one of the cornerstone surgical treatments for renal tumors and can be performed via open, laparoscopic, or robotic approaches. Despite advances in surgical techniques, postoperative complications continue to represent a significant clinical concern [1]. These complications include perioperative mortality, pain, bleeding, infection, ileus, acute thromboembolic and cardiovascular events, prolonged hospitalization, the need for intensive care, and increased healthcare costs [2, 3].

In high-risk surgeries such as radical nephrectomy, accurate preoperative risk assessment is crucial for informed decision-making by both patients and surgeons. This approach enables early detection of potential complications, optimization of treatment planning, and more reliable prognostic prediction. Imaging-based preoperative scoring systems have been reported to contribute to complication risk estimation by providing a more precise characterization of tumor complexity [4].

In recent years, it has been increasingly emphasized that outcomes following kidney tumor surgery are influenced not only by surgical technique but also by patient-related systemic factors. In particular, advanced age and frailty have been shown to play a critical role in postoperative morbidity, in conjunction with the patient’s overall physiological reserve [5]. Furthermore, recent analyses based on large-scale databases have demonstrated that major cardiovascular events occur at a considerable rate following radical nephrectomy, and that this risk is associated not only with the surgical procedure itself but also with multiple factors, including age, comorbidities, and overall health status [6]. These findings support the need for a multidimensional approach to surgical risk assessment.

Scoring systems developed to predict surgical risk were initially designed for heterogeneous populations with diverse pathologies but were later applied to patients undergoing specific diagnoses or single surgical procedures. The Estimation of Physiologic Ability and Surgical Stress (E-PASS) score is a system originally developed to predict postoperative complication risk in patients undergoing elective gastrointestinal surgery that integrates both preoperative physiological status and intraoperative variables [7]. Subsequently, the modified version, mE-PASS, introduced by Haga et al., simplified data entry by using fixed coefficients for surgical stress and demonstrated nationwide validity [8]. The Physiological and Operative Severity Score for the Enumeration of Mortality and Morbidity (POSSUM), developed by Copeland et al., consists of two components: physiological (PS) and operative severity (OS) variables [9]. This model has been proposed as a suitable risk assessment tool for general surgery [10]. Utilizing scores derived from twelve physiological and six operative variables, it was developed to predict in-hospital mortality and postoperative morbidity. The physiological variables include age, cardiovascular and respiratory status, as well as laboratory parameters, whereas the operative variables encompass factors such as the magnitude of surgery, intraoperative blood loss, and operative severity. However, POSSUM has been reported to overestimate postoperative mortality, particularly in low-risk patients, leading Whiteley et al. to develop the Portsmouth modification (P-POSSUM) [11].

In recent years, several studies have comparatively evaluated the effectiveness of these three scoring systems in predicting postoperative complications within the field of general surgery [12, 13]. In urologic surgery, however, the POSSUM model has been used predominantly to predict morbidity and mortality following radical cystectomy, whereas studies assessing complications after nephrectomy have been limited mostly to the E-PASS system [14–16]. To the best of our knowledge, the mE-PASS score has not yet been evaluated in urologic surgeries, and no study to date has compared the mE-PASS score with the E-PASS score and POSSUM score in patients undergoing radical nephrectomy.

In addition, preoperative biomarkers reflecting the systemic inflammatory response, immune status, and nutritional condition play important roles in surgical risk assessment. Among these, the modified Glasgow prognostic score (mGPS), prognostic nutritional index (PNI), and neutrophil‒lymphocyte ratio (NLR) have been shown to be associated with both postoperative complications and overall prognosis [17–19].

The aim of this study was to compare the discriminative performance of the E-PASS, mE-PASS, and POSSUM scoring systems in predicting postoperative complications in patients undergoing radical nephrectomy for renal tumors. As a secondary objective, the association of mGPS, PNI, and NLR with the development of postoperative complications was also evaluated.

## Materials and methods

**Fig. 1:**
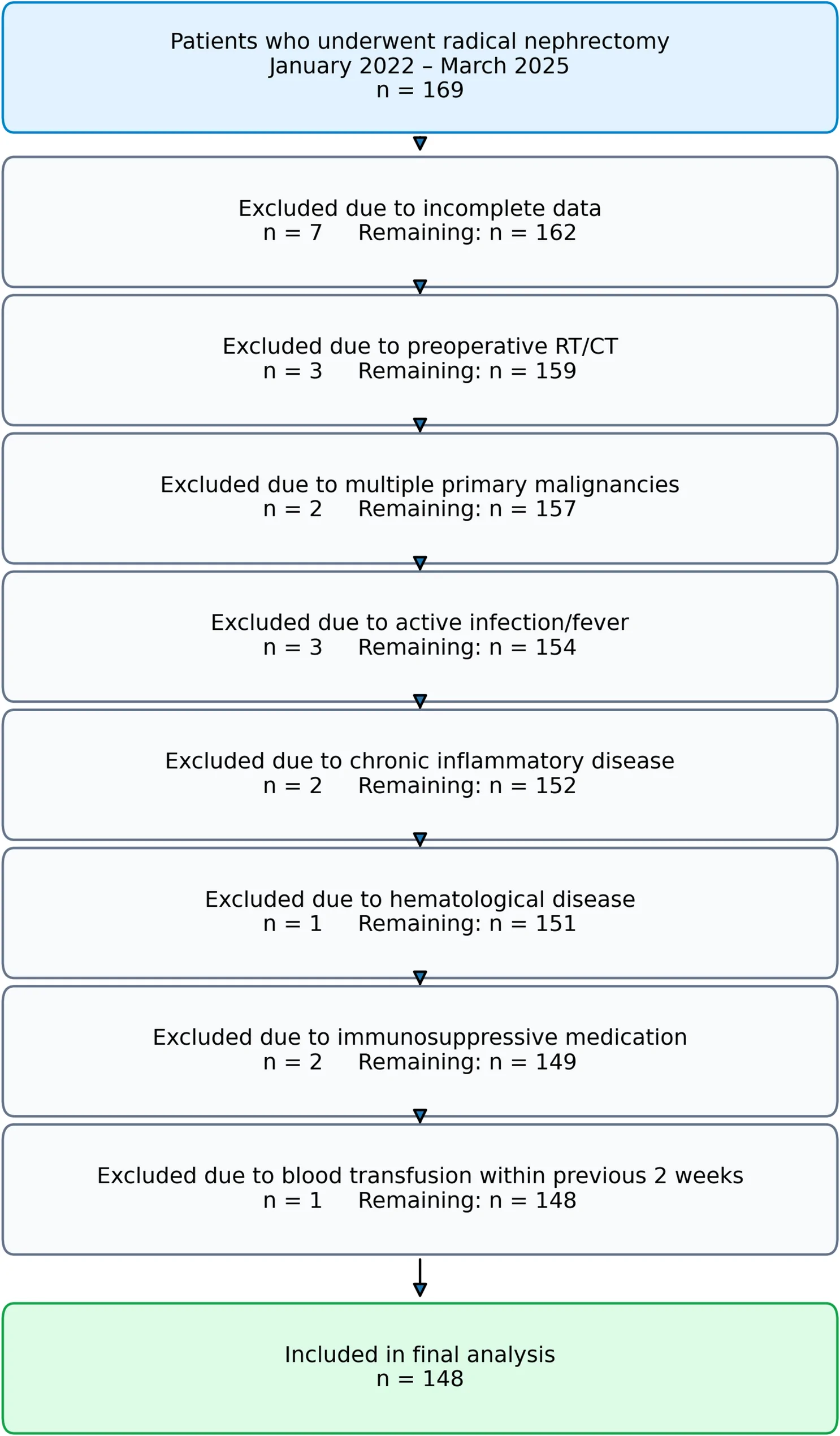
Flowchart of patient selection and study design

This retrospective study included patients who underwent radical nephrectomy in the urology clinic between January 2022 and March 2025 and had a histopathological diagnosis of a renal tumor. Physiological and surgical parameters were retrospectively obtained from the hospital information system, anesthesia records, and perioperative nursing notes. The use of patient data was approved by the institutional ethics committee (Date/Reference No.: 08.05.2025–13/497). The study was conducted in accordance with the principles of the Declaration of Helsinki, and due to the purely observational nature of the data collection process, informed consent was not obtained. Patients who were not suitable for partial nephrectomy and therefore underwent radical nephrectomy based on tumor size, location, surgical complexity, and patient-related clinical factors were included in the study. Patients with missing data for any variable required to calculate the scoring systems were excluded from the analysis, and only those with complete datasets were evaluated. In addition, 21 patients were excluded due to preoperative radiotherapy or chemotherapy for any indication, the presence of multiple primary malignancies, active infection or fever, chronic inflammatory or hematological disease, use of immunosuppressive therapy, or a history of blood transfusion within the preceding two weeks. These patients were excluded to minimize potential confounding effects on postoperative outcomes and systemic inflammatory markers. The patient selection process and study flow are illustrated in Fig. 1.

Preoperative, intraoperative, and postoperative data of all patients were reviewed. The analyzed variables included age, sex, body mass index (BMI, kg/m²), presenting symptoms, history of previous abdominal surgery, lesion laterality and size, American Society of Anesthesiologists (ASA) physical status classification, Eastern Cooperative Oncology Group (ECOG) performance status, neutrophil‒lymphocyte ratio (NLR), platelet‒lymphocyte ratio (PLR), serum albumin level, comorbidities, and type of surgery. In addition, the operative time, estimated blood loss, need for transfusion, incision length, incidence of postoperative complications, and length of hospital stay were recorded. Postoperative complications were assessed according to the Clavien–Dindo classification, and complications of grade ≥ 2 were included in the analysis. It has been reported that interobserver agreement is limited in the assessment of low-grade complications (particularly grade 1), which may result in variability in their reporting [20]. In this context, some studies have defined major complications as Clavien grade ≥ 3 [21] whereas others have analyzed complications of grade ≥ 2 [12, 22–24]. Therefore, in the present study, major complications were defined as Clavien grade ≥ 2. Morbidity related to postoperative complications was defined as events occurring within 30 days after surgery.

### E-PASS system

The E-PASS scoring system was developed to estimate surgical risk and consists of three components: the preoperative risk score (PRS), the surgical stress score (SSS), and the comprehensive risk score (CRS) [5 > 7]. For each patient in the present study, PRS, SSS, and CRS values were calculated. The formulas used in the E-PASS system are presented in Supplementary Table 1.

### mE-PASS system

To determine the mE-PASS score, the preoperative risk score (PRS) was calculated via the same equation as in the original E-PASS system. In the mE-PASS model, a fixed surgical stress score (SSSf) of 0.11 was applied for radical nephrectomy, representing the median SSS value obtained in the original E-PASS system (Supplementary Table 1) [8].

### POSSUM system

A total of 18 variables were used to calculate the physiological score (PS) and operative severity score (OS). The PS included age; cardiac and respiratory findings; pulse rate; systolic blood pressure; Glasgow Coma Scale (GCS) score; and serum urea, potassium, sodium, and hemoglobin levels; white blood cell counts; and electrocardiogram findings. Unless otherwise specified, all patients were assumed to have a GCS of 15 preoperatively. The OS included the magnitude of the operation, operative timing, amount of blood loss, degree of peritoneal contamination, presence of malignancy, and total number of procedures performed. Each variable was categorized into four levels (1, 2, 4, or 8 points), with higher scores indicating greater risk. The POSSUM morbidity rate (R) was calculated by incorporating the PS and OS values into regression equations (Supplementary Table 1) [9].

### Additional prognostic parameters

The modified Glasgow prognostic score (mGPS) is a prognostic indicator reflecting the preoperative systemic inflammatory response and is calculated on the basis of C-reactive protein (CRP) and serum albumin levels (Supplementary Table 1) [25]. The prognostic nutritional index (PNI) is a simple preoperative marker used to assess immune and nutritional status and is based on the serum ALB concentration and total lymphocyte count (Supplementary Table 1) [18]. The neutrophil-to-lymphocyte ratio (NLR), a prognostic biomarker reflecting systemic inflammation and immunosuppression, was calculated by dividing the absolute neutrophil count by the lymphocyte count [19]. Preoperative neutrophil and lymphocyte values were obtained from complete blood counts performed within 24 h prior to surgery. These parameters were evaluated as part of a secondary analysis to assess their association with the development of postoperative complications.

### Surgical technique

Open nephrectomies were performed via a retroperitoneal approach, whereas laparoscopic nephrectomies were carried out via a transperitoneal route. Standard surgical principles were followed in all patients.

### Statistical analysis

Continuous variables are expressed as the means ± standard deviations or medians (interquartile ranges, IQRs), whereas categorical variables are presented as numbers and percentages (n, %). The normality of the data distribution was assessed via the Kolmogorov–Smirnov test. Comparisons between groups were performed via Student’s *t* test or the Mann–Whitney *U* test for continuous variables and the chi-square test for categorical variables. The sensitivity, specificity, and predictive performance of the scoring systems were evaluated via receiver operating characteristic (ROC) curve analysis, and the area under the ROC curve (AUC) values were calculated. Statistical analyses were performed via SPSS version 26.0 (IBM Corp., Armonk, NY, USA). A *p* value of < 0.05 was considered to indicate statistical significance.

## Results

The mean age of the 148 patients who underwent radical nephrectomy was 62.4 ± 12.8 years, and 86 patients (58.1%) were male. The mean body mass index (BMI) was 28.1 ± 4.2 kg/m². There were no significant differences between patients with and without postoperative complications in terms of age, sex, or BMI (Table 1).

**Table 1:** Baseline clinicopathological characteristics of patients with and without postoperative complications

	Total (*n* = 148)	Non-complications (*n* = 97, 65.5%)	Complications (*n* = 51, 34.5%)	*P*
Age (years, mean ± SD)	62.36 ± 12.78	61.71 ± 12.83	63.59 ± 12.70	0.398
Gender				0.104
Male	86 (58.1%)	61 (62.9%)	25 (49.0%)	
Female	62 (41.9%)	36 (37.1%)	26 (51.0%)	
BMI (kg/m², mean ± SD	28.06 ± 4.22	27.85 ± 4.30	28.47 ± 4.07	0.389
Presenting symptoms	148 (100%)			**0.011**
Incidental finding, n (%)	84 (56.7%)	62 (63.9%)	22 (43.1%)	
Flank pain, n (%)	37 (25.0%)	22 (22.7%)	15 (29.4%)	
Hematuria, n (%)	22 (14.9%)	13 (13.4%)	9 (17.6%)	
Mass, n (%)	4 (2.7%)	0 (0%)	4 (7.8%)	
Renal bleeding, n (%)	1 (0.7%)	0 (0%)	1 (2.0%)	
History of previous surgery, n (%)	63 (42.6%)	38 (39.2%)	25 (49.0%)	0.250
ASA score				0.212
Class 1, n (%)	5 (3.4%)	4 (4.1%)	1 (2.0%)	
Class 2, n (%)	76 (51.4%)	55 (56.7%)	21 (41.2%)	
Class 3, n (%)	60 (40.5%)	33 (34.0%)	27 (52.9%)	
Class 4, n (%)	7 (4.7%)	5 (5.2%)	2 (3.9%)	
Performance status				0.325
Level 0, n (%)	63 (42.6%)	45 (46.4%)	18 (35.3%)	
Level 1, n (%)	55 (37.2%)	35 (36.1%)	20 (39.2%)	
Level 2, n (%)	21 (14.2%)	13 (13.4%)	8 (15.7%)	
Level 3, n (%)	9 (6.1%)	4 (4.1%)	5 (9.8%)	
Severe cardiac disease, n (%)	19 (12.8%)	10 (10.3%)	9 (17.6%)	0.192
Severe pulmonary disease, n (%)	17 (11.5%)	6 (6.2%)	11 (21.6%)	**0.006**
Hemoglobin (g/dL, mean ± SD)	12.54 ± 1.94	12.80 ± 1.73	12.06 ± 2.22	**0.024**
NLR (mean ± SD)	3.89 ± 2.77	3.47 ± 2.51	4.62 ± 3.06	**0.030**
PLR (mean ± SD)	158.2 ± 74.9	152.4 ± 71.1	168.3 ± 81.3	0.218
PNI (mean ± SD)	45.3 ± 5.5	46.2 ± 5.2	43.5 ± 5.6	**0.004**
mGPS (mean ± SD)	0,82 ± 0,87	0,73 ± 0,83	1 ± 0,91	0.075
Pathology				0.130
Benign renal tumors, n (%)	13 (%8,8)	11 (%11,3)	2 (%3,9)	
Malignant renal tumors, n (%)	135%91,2)	86 (%88,7)	49 (%96,1)	
Lesion diameter (mm, mean ± SD)	67.2 ± 34.1	61.5 ± 30.6	76.5 ± 37.2	**0.009**
Type of nephrectomy				**< 0.001**
Open, n (%)	108 (73.0%)	61 (62.9%)	47 (92.2%)	
Laparoscopic, n (%)	40 (27.0%)	36 (37.1%)	4 (7.8%)	
Hospital stay (days, mean ± SD)	5.0 ± 3.9	4.1 ± 2.6	6.7 ± 5.0	**< 0.001**
Operation time (min, mean ± SD)	142.8 ± 44.2	139.1 ± 42.6	149.2 ± 46.3	0.256
Bleeding volume (ml, mean ± SD)	230.7 ± 201.4	154.2 ± 136.3	361.5 ± 243.8	**< 0.001**
E-PASS PRS (Median) (IQR)	0.47 [0.33–0.69]	0.44 [0.30–0.63]	0.53 [0.37–0.77]	**0.018**
E-PASS SSS (Median) (IQR)	0.07 [− 0.11–0.21]	-0.05 [− 0.12–0.06]	0.20 [0.08–0.26]	**< 0.001**
E-PASS CRS (Median) (IQR)	0.18 [0.00–0.49]	0.10 [− 0.02–0.31]	0.33 [0.21–0.64]	**< 0.001**
mE-PASS (CRSf) (Median) (IQR)	0.43 [0.36–0.52]	0.41 [0.34–0.51]	0.46 [0.38–0.60]	**0.027**
POSSUM (Median) (IQR)	28.5 [16.2–45.3]	18.6 [13.1–29.5]	41.0 [23.1–61.7]	**< 0.001**
POSSUM PS (Median) (IQR)	4.12 [1.90–10.23]	2.71 [1.30–5.38]	6.92 [2.98–15.88]	**< 0.001**
POSSUM OS (Median) (IQR)	1.31 [0.58–2.80]	0.70 [0.33–1.51]	2.22 [1.02–5.51]	**< 0.001**

*BMI* Body Mass Index, *ASA* American Society of Anesthesiologists, *NLR* Neutrophil-to-lymphocyte ratio, *PLR* Platelet-to-lymphocyte ratio, *PNI* Prognostic Nutritional Index, *E-PASS* Estimation of Physiologic Ability and Surgical Stress, *PRS* Preoperative Risk Score, *SSS* Surgical Stress Score, *CRS* Comprehensive Risk Score, *mE-PASS* Modified Estimation of Physiologic Ability and Surgical Stress, *POSSUM* Physiological and Operative Severity Score for the enUmeration of Mortality and Morbidity, *PS* Physiological Score, *OS* Operative Severity Score, *IQR* Interquartile Range. Bold values indicate statistical significance (*p* < 0.05)

With respect to the mode of clinical presentation, incidentally detected tumors were more common in the group without complications, whereas presentation with a palpable mass or bleeding was more common among patients who developed complications (*p* < 0.05). There were no significant differences between the groups in terms of previous abdominal surgery or ASA classification (Table 1).

In the complication group, the incidence of severe pulmonary disease was greater. Moreover, patients in this group had lower preoperative hemoglobin levels, higher neutrophil‒lymphocyte ratios (NLRs), and lower prognostic nutritional index (PNI) values. The tumor size and rate of open surgery were also greater in the complication group. Additionally, the hospital stay was longer, and intraoperative blood loss was greater in this group (*p* < 0.05) (Table 1).

Among the patients, 108 (73.0%) underwent open nephrectomy, while 40 (27.0%) underwent laparoscopic nephrectomy. Benign renal tumors were identified in 13 patients (8.8%) (including 6 oncocytomas, 5 angiomyolipomas, 1 cystadenoma, and 1 schwannoma), whereas 135 patients (91.2%) had malignant renal tumors (renal cell carcinoma) (Table 1).

The E-PASS scoring components—preoperative risk score (PRS), surgical stress score (SSS), and comprehensive risk score (CRS)—as well as the mE-PASS (CRSf) score and the POSSUM components (physiological score [PS] and operative severity score [OS]) together with the POSSUM morbidity risk were significantly greater in the complication group (*p* < 0.05) (Table 1).

Postoperative complications occurred in 51 patients (34.5%), with the most common being bleeding requiring transfusion, which was observed in 36 patients (70.5%). In addition, 2 patients (3.9%) experienced postoperative mortality (Table 2).

**Table 2:** Postoperative complications according to the Clavien–Dindo classification

Grade	Definition	*n* (%)
Grade 1	Any deviation from the normal postoperative course without the need for pharmacological treatment or surgical, endoscopic, and radiological interventions Allowed therapeutic regimens are the following: drugs as antiemetics, antipyretics, analgetics, diuretics, electrolytes, and physiotherapy. This grade also includes wound infections opened at the bedside	
Grade 2	Requiring pharmacological treatment with drugs other than such allowed for grade I complications Bleeding requiring blood transfusion	36 (70,5)
Grade 3	Complications requiring surgical, endoscopic or radiological intervention	
Grade 3a	Complications that do not require general anesthesia Wound infection requiring non-anesthetic for intervention Wound site defect requiring intervention without anesthesia Bleeding/hematoma requiring intervention without anesthesia	4 (7,7) 1 (2) 1 (2)
Grade 3b	Complications requiring general anesthesia Small bowel perforation Ileus Liver laceration	2 (3,9) 1 (2) 1 (2)
Grade 4	Complications that are life-threatening and require intensive care	
Grade4a	Single organ failure Cerebrovascular event Pulmonary embolism	1 (2) 1 (2)
Grade 4b	Multiple organ failure Urosepsis	1 (2)
Grade 5	Death of a patient	2 (3,9)

**Fig. 2:**
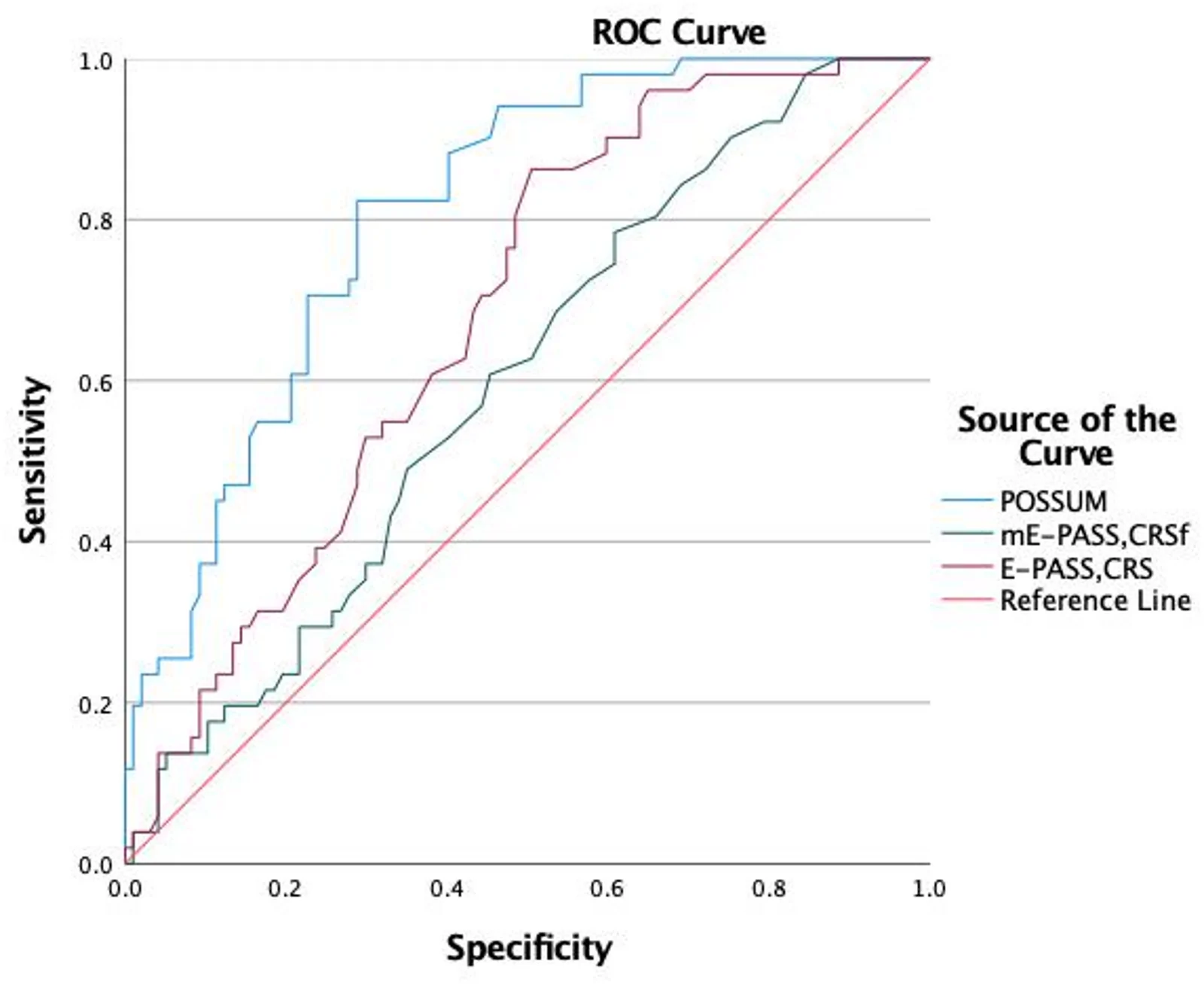
ROC curves of the POSSUM, E-PASS and mE-PASS for the prediction of early postoperative complications after nephrectomy

The discriminative performance of the three scoring systems was evaluated via receiver operating characteristic (ROC) curve analysis, expressed as the area under the curve (AUC). Among them, the POSSUM scoring system demonstrated the highest AUC value for predicting postoperative complications (0.810; 95% CI: 0.742–0.878; *p* < 0.001). The POSSUM score showed a balanced predictive performance, with 82.4% sensitivity and 71.1% specificity, indicating good accuracy in predicting postoperative complications (Fig. 2; Table 3).

**Table 3:** Efficacy of each scoring system in predicting early complications after nephrectomy

	AUC	The optimal cutoff value (sensitivity, specificity)	95% CI	*p*
POSSUM	0.810	0,22 (0.824, 0.711)	0.742–0.878	**< 0.001**
E-PASS (CRS)	0.682	0,15 (0.863, 0.505)	0.597–0.767	**< 0.001**
mE-PASS (CRSf)	0.599	0,42 (0.608, 0.546)	0.506–0.692	0.048

*AUC* Area Under the Curve, *POSSUM* Physiological and Operative Severity Score for the enUmeration of Mortality and Morbidity, *E-PASS* Estimation of Physiologic Ability and Surgical Stress, *CRS* Comprehensive Risk Score, *mE-PASS* Modified Estimation of Physiologic Ability and Surgical Stress. Bold values indicate statistical significance (*p* < 0.05)

The E-PASS score also demonstrated a statistically significant but more limited predictive performance (AUC: 0.682; 95% CI: 0.597–0.767; *p* < 0.001). In contrast, the mE-PASS (CRSf) score exhibited a notably lower discriminative ability (Fig. 2; Table 3).

Among the evaluated models, the POSSUM score was identified as the most useful tool for predicting postoperative complications following radical nephrectomy.

## Discussion

A clear understanding of postoperative complications in urologic surgeries is essential for improving the overall quality of care and patient management. Therefore, it is recommended that complications be standardized and reported and that reliable risk prediction tools be utilized [26]. Although there is no urology-specific risk assessment tool, several scoring systems have been applied in this field [27]. In particular, systems such as R.E.N.A.L., Preoperative Aspects and Dimensions Used for an Anatomical (PADUA), and Diameter–Axial–Polar (DAP) have been developed to predict perioperative outcomes after partial nephrectomy, focusing solely on tumor-related anatomical features [28, 29]. However, these models do not consider intraoperative variables, which can significantly influence surgical outcomes. In recent years, interest in developing risk models that integrate both preoperative and intraoperative variables in urologic surgeries has increased [14–16, 30].

To the best of our knowledge, this study is the first to compare the ability of the E-PASS, mE-PASS, and POSSUM scoring systems to predict postoperative complications in urologic surgeries, particularly radical nephrectomy, by simultaneously incorporating both preoperative and intraoperative variables.

### Perioperative risk scoring systems

The E-PASS, mE-PASS, and POSSUM scoring systems were originally developed in the field of general surgery, and their performance in predicting postoperative complications and mortality has been investigated across various organs and surgical procedures. In a study involving patients who underwent pancreatic resection, all three scores demonstrated moderate predictive power for postoperative morbidity and mortality [31]. In contrast, in hepatic surgery, these models reportedly have high discriminative ability [32]. Following laparoscopic radical gastrectomy, the mE-PASS score had the highest AUC value (0.846), whereas the POSSUM score had the lowest predictive performance (AUC: 0.648) [12]. In biliary tract cancer surgery, both the mE-PASS and POSSUM scoring systems were found to be ineffective in predicting postoperative morbidity among patients over 70 years of age. In contrast, the E-PASS score, particularly when combined with perioperative parameters, demonstrated greater accuracy in predicting severe complications [13]. Similarly, in a study comparing the POSSUM, P-POSSUM, and E-PASS scores in patients who underwent hilar cholangiocarcinoma surgery, all three models effectively predicted postoperative morbidity and mortality. However, only the POSSUM score (AUC: 0.843) showed superior performance in predicting postoperative morbidity following high-severity procedures [33].

In the field of urologic surgery, the performance of the POSSUM and E-PASS scoring systems has been evaluated separately in several studies. The POSSUM and Portsmouth-POSSUM (P-POSSUM) scores have been reported to be effective tools for predicting morbidity and mortality risk following radical cystectomy [34]. However, in a comprehensive literature review, Razdan et al. concluded that these models possess limited predictive accuracy [14]. Conversely, the E-PASS score has been shown to be an effective and practical system for predicting postoperative complications after both laparoscopic nephrectomy and robot-assisted partial nephrectomy [15, 16]. In our study, the POSSUM score achieved the highest AUC value (0.810) for the prediction of postoperative complications following radical nephrectomy. The E-PASS score, although consistent with previous studies reporting its usefulness as a standalone predictor, demonstrated a more limited predictive performance (AUC = 0.682). This may be attributed to the fact that the POSSUM system provides a more comprehensive assessment by incorporating both physiological and operative parameters. Moreover, physiology-weighted scoring systems are thought to be more sensitive in predicting systemic complications. However, it should be acknowledged that neither the E-PASS nor the POSSUM system is based exclusively on preoperative variables. Both models incorporate intraoperative parameters, including surgical stress-related factors and blood loss. Therefore, these scoring systems may be more appropriately interpreted as perioperative risk stratification tools rather than purely preoperative prediction models. Furthermore, the inclusion of intraoperative variables that are closely related to postoperative outcomes may partly explain the superior predictive performance of the POSSUM score observed in the present study. Nevertheless, since the AUC did not exceed 0.9, the findings should be validated in larger multicenter studies.

### Preoperative biomarkers and risk factors

On the other hand, surgical risk is not limited to physiological and intraoperative parameters; the patient’s systemic inflammatory response, nutritional status, and immune capacity also play crucial roles in determining postoperative outcomes. Studies in patients with renal tumors have reported that low prognostic nutritional index (PNI) values are associated with higher rates of postoperative complications and reduced survival [18, 35]. In our study, consistent with the literature, the PNI values were significantly lower in the complication group. These findings suggest that low PNI levels, which reflect poor immune and nutritional status, may serve as important determinants of postoperative complications, particularly in high-stress procedures such as radical nephrectomy. In parallel with our results, the neutrophil-to-lymphocyte ratio (NLR) is another important prognostic marker that reflects systemic inflammation and related immunosuppression. Elevated preoperative NLR values have been linked to an increased risk of postoperative complications and poor prognosis [19, 36]. In our study, preoperative NLR values were significantly greater in patients who developed complications. This finding suggests that elevated preoperative NLR may serve as an important predictor of postoperative complications following radical nephrectomy, possibly through mechanisms involving increased inflammatory burden and immune dysregulation [19, 36].

Similarly, in our study, presentation with a palpable mass or bleeding was more common among patients who developed complications. Henderson et al. reported that hematuria was a predictor of major postoperative complications following nephrectomy [37]. This finding suggests that symptomatic presentations may be associated with greater surgical complexity and an increased risk of complications. Furthermore, in the complication group, the length of hospital stay, amount of blood loss, tumor size, and rate of open surgery were significantly greater, whereas preoperative hemoglobin levels were lower. These results are consistent with previously reported findings in the literature [37, 38]. Finally, the higher frequency of severe pulmonary disease among patients with complications aligns with the inclusion of this comorbidity as a preoperative variable in both the POSSUM and E-PASS scoring systems.

### Limitations

This study has several limitations. First, the lack of nephrometry scores (e.g., RENAL, PADUA), which provide a more objective assessment of tumor complexity, may have limited a more comprehensive evaluation of the impact of tumor characteristics on surgical outcomes. Second, the single-center, retrospective design may limit the generalizability of the findings and introduce selection bias. In addition, factors such as institutional surgical volume, surgeon experience, and perioperative management protocols may have influenced the outcomes. Third, the evaluation of only 30-day postoperative complications and the inclusion of Clavien grade ≥ 2 complications in the analysis may have affected the interpretation of the results. Furthermore, the combined analysis of open and laparoscopic nephrectomy cases may limit the interpretation of the findings, given the differences in complication profiles between these surgical approaches. Finally, the absence of multivariable analysis and external validation represents an important limitation of the present study. In addition, some intraoperative components of the POSSUM score, particularly estimated blood loss, may be closely related to postoperative outcomes such as transfusion-requiring complications. Therefore, part of the predictive performance of POSSUM may reflect the incorporation of perioperative events rather than purely preoperative risk estimation. This consideration should be taken into account when interpreting the findings.

## Conclusion

The POSSUM and E-PASS scoring systems may be considered as useful tools for predicting postoperative complications following radical nephrectomy. In our study, the POSSUM score demonstrated the highest predictive performance, while the E-PASS score showed a more limited but statistically significant discriminative ability. In contrast, the lower performance of the mE-PASS score may be related to its simplified structure, including the use of fewer variables and the representation of certain parameters with fixed coefficients. Additionally, low PNI and high NLR values were found to be associated with the development of complications. Our findings suggest that POSSUM represents a clinically applicable and practical perioperative risk stratification model for assessing the risk of postoperative complications following radical nephrectomy, while immune-nutritional parameters such as the PNI and NLR may serve as complementary markers in surgical risk prediction.However, since none of the evaluated scoring systems achieved an AUC greater than 0.9, larger multicenter studies are warranted to increase the accuracy and generalizability of these predictive models.

## Supplementary Information

Supplementary Material 1.

## Data Availability

The full dataset generated and analyzed during the current study are available from the corresponding author on reasonable request.
