# Supplementary material for: Evaluation of the E-PASS, mE-PASS, and POSSUM scoring systems for predicting postoperative complications following radical nephrectomy
Source: BMC Surg. 2026 Jun 16;26:566. doi: 10.1186/s12893-026-03960-7 (PMC13501659; doi:10.1186/s12893-026-03960-7)
Supplement: Supplementary file 1 [file 12893_2026_3960_MOESM1_ESM.docx]

**Supplementary Table 1: Summary of scoring systems and calculation formulas**

| **Score** | **Formula** |
| --- | --- |
| **E-PASS, PRS** | PRS= -0,0686 + 0,00345X1 + 0,323X2 + 0,205X3 + 0,153X4 + 0,148X5 + 0,0666X6  (where X1 represents age, X2 represents patients with or without severe heart disease (1 or 0 points), X3 represents patients with or without severe pulmonary disease (1 or 0 points), X4 represents patients with or without diabetes mellitus (1 or 0 points), X5 represents the index of performance condition (range, 0–4 points), and X6 represents the American Society of Anesthesiologists (ASA) score (range, 1–5 points).  Severe heart disease was considered as New York Heart Association (NYHA) grade 3/4 or serious cardiac asthma needing mechanical assistance. Serious pulmonary illness was classified as a vital capacity of less than 60% or a first second of forced expiratory volume of less than 50% ) |
| **E-PASS, SSS** | SSS=-0,342 + 0,0139X1 + 0,0392X2 + 0,352X3  (X1 blood loss/body weight (g/kg), X2 operating time (hour), X3 skin incision length (0: minor incision, 1: laparotomy or thoracotomy only, 2: laparotomy and thoracotomy ) |
| **E-PASS, CRS** | CRS= -0,328 + 0,936 (PRS) + 0,976 (SSS) |
| **mE-PASS** | mE-PASS (CRSf) = 0.052 + 0.58(PRS) + 0.83(SSSf)  (CRSf : Fixed Comprehensive Risk Score , SSSf: Fixed Surgical Stress Score ) |
| **POSSUM** | POSSUM Morbidity Risk = ln[R/(1−R)] = −5.91 + (0.16 × PS) + (0.19 × OS)  (where R is the predicted risk of morbidity, PS: Physiological Score, OS:Operative Severity Score) |
| mGPS | CRP ≥10 mg/L and albumin <35 g/L = 2; CRP ≥10 mg/L alone = 1; normal CRP = 0.  (CRP: C-reactive protein) |
| **PNI** | PNI: 10 × serum albumin (g/dL) + 0.005 × total lymphocyte count (/mm³) |

*E-PASS: Estimation of Physiologic Ability and Surgical Stress, PRS: Preoperative Risk Score, SSS: Surgical Stress Score, CRS: Comprehensive Risk Score, mE-PASS: modified Estimation of Physiologic Ability and Surgical Stress, m POSSUM: Physiological and Operative Severity Score for the enUmeration of Mortality and Morbidity, mGPS: modified Glasgow Prognostic Score, PNI: Prognostic Nutritional Index*
